# Mid-term outcomes of a stemless ceramic head anatomic total shoulder replacement

**DOI:** 10.1186/s12891-021-04988-x

**Published:** 2022-01-15

**Authors:** Maciej J. K. Simon, Jennifer A. Coghlan, Jeff Hughes, Warwick Wright, Richard J. Dallalana, Simon N. Bell

**Affiliations:** 1 Melbourne Shoulder and Elbow Centre, 1/80 Beach Road, Sandringham, VIC 3191 Australia; 2 University Medical Center Schleswig-Holstein, Campus Kiel, Department of Orthopaedics and Trauma Surgery, Arnold-Heller-Strasse 3, 24105 Kiel, Germany; 3 Department of Surgery, (School of Clinical Sciences, Monash Health) Monash University, Monash University, Melbourne, VIC Australia; 4 Sydney Shoulder and Elbow Associates, Chatswood, Sydney, Australia; 5 Malvern Orthopaedic Centre, Melbourne, Australia; 6 Park Clinic Orthopaedics, East Melbourne, Melbourne, Australia

**Keywords:** Anatomic total shoulder replacement, Stemless, Ceramic head prosthesis, Clinical scores, Lazarus score

## Abstract

**Background:**

In an anatomic shoulder replacement (aTSR) good results have been reported with the use of a stemless humeral prosthesis. In vitro a ceramic articulation with polyethylene has been shown to produce less polyethylene wear particles than with metal. This study aims to evaluate clinical and radiographic results of a stemless aTSR with a ceramic head articulating with a polyethylene glenoid component, with mid-term follow-up.

**Methods:**

All patients (*n* = 92) in this prospective study had an aTSR utilizing a stemless humeral component with a ceramic head and a cemented double pegged cemented polyethylene glenoid component for glenohumeral osteoarthritis. Pre- and postoperative clinical evaluations at 2 years were performed using the ASES score, Constant score, SPADI score, DASH score, VAS pain score, patient satisfaction and range of motion. There was a 5-year evaluation of SPADI, ASES, pain, and satisfaction, plus radiographic assessment of glenoid component radiolucent lines and humeral osteolysis.

**Results:**

Seventy-four cases (68.1 ± 7.1 years) had a five-year follow-up and demonstrated active elevation improvement from 91.3° preoperatively to 151.1° (*p* < 0.001). Further improvement was identified with the ASES from 41.6 to 94.3, the SPADI from 62.9 to 4.3, VAS pain from 5.6 to 0.4 (0–10), and satisfaction levels were at 96%. Sixty-two cases had no glenoid radiolucent lines with a maximum Lazarus score of 2 in one patient. Constant scores, available up to 2 years, improved significantly from 30.3 to 77.9 (*p* < 0.001). There was one case that required revision for glenoid loosening.

**Conclusions:**

Overall, the 5-year results of this ceramic head prosthesis demonstrated good radiographic and clinical outcomes.

**Trial registration:**

ACTRN12613001183774. Registered: 29 October 2013 - Retrospectively registered.

Australian New Zealand Clinical Trials Registry (ANZCTR).

## Background

If symptomatic glenohumeral osteoarthritis of the shoulder fails to respond to conservative measures, and patients have an intact functioning rotator cuff, they could be suitable for an anatomic total shoulder replacement [1].

There has been development over the past decade in anatomic components for both the humerus and the glenoid [2, 3]. The humeral stemless anatomic total shoulder replacement (aTSR) has gained importance in the past decade as it recreates the native humeral anatomy and preserves bone stock with less implant material, and if needed, revision surgery of the humeral component is easier than with a stemmed prosthesis [4, 5]. A stemless aTSR also allows for the use of a ceramic head, which in vitro produces less polyethylene wear than a metal head when articulating with a polyethylene glenoid component and in vivo less osteolysis [6, 7].

The prosthesis used in the current study is the most common elective stemless aTSR used in the UK [8]. A 2-year follow-up of 12 patients, and another 4-year follow-up study for this type of prosthesis showed promising results [9, 10]. This prospective study analyses the results of a stemless humeral component with a ceramic head, which articulates with a cemented polyethylene glenoid component. The aim of this 5-year mid-term multicentre follow-up study is to evaluate patient-reported outcome measures (PROMs) and radiographic results of this stemless aTSR.

## Methods

### Study design and ethics approval

The study was a prospective clinical, multicentre study with a 5-year follow-up.

The current study is a continuation of the 2-year follow-up study published in 2014, with greater numbers and longer follow up [9]. The primary goal of this mid-term follow-up study is to examine clinical outcomes (PROM’s, ROM, VAS, satisfaction) and radiographic results of this stemless aTSR.

Five senior orthopaedic surgeons performed the surgical procedures in this study. All shoulders were replaced with an anatomic TSR, the stemless Affinis® Short (Mathys AG, Bettlach, Switzerland) humeral component and a double pegged cemented all polyethylene glenoid component made of standard, not cross-linked, polyethylene.

The study received ethics approval from the Monash University (CF10/0376–2,010,000,170) and was registered at the Australian New Zealand Clinical Trials Registry (ANZCTR) ACTRN12613001183774. The study was performed in accordance with the latest version of the Declaration of Helsinki.

### Eligibility, inclusion and exclusion criteria

Consecutive patients with advanced primary osteoarthritis of the glenohumeral joint and an intact rotator cuff in a patient who has failed conservative measures and considered their symptoms were severe enough to have a replacement were eligible to be enrolled in this study. The exclusion criteria were age over 80 years, inability to replace the glenoid, severe osteoporosis, rotator cuff tear, post fracture/traumatic osteoarthritis or proximal humeral deformity, abnormal neurology, or inability to comply with the study requirements.

The rotator cuff was assessed with a clinical examination. Additionally, radiological imaging including CT scans were obtained for all cases, which assisted assessing the rotator cuff. If there remained any doubt, a MRI scan was organized to confirm the rotator cuff integrity. If the rotator cuff was not intact, the patient was excluded. Glenoid assessment was carried out preoperatively with a CT scan in regards to version, bone loss and glenoid type. If the morphology of the glenoid was judged by the surgeon unsuitable for adequate implantation of the double pegged cemented all polyethylene glenoid component, the patient was deemed unsuitable for implantation of the stemless Affinis® Short (Mathys AG, Bettlach, Switzerland) and hence excluded from the study. Osteoporosis assessment was carried out with pre-operative radiological imaging and intraoperatively after removal of the humeral head. However, there were no patients excluded from implantation of this prosthesis related to pre-operative radiology or intraoperative judgement of osteoporosis.

### Surgical technique and postoperative rehabilitation

The operations were performed in all cases through a deltopectoral approach with tenodesis of the biceps tendon and mobilization of the subscapularis with either a tenotomy or a lesser tuberosity osteotomy. Capsular release was then performed. The humeral head was resected anatomically using the provided guides [9].

Glenoid preparation was then performed in a standard manner for the Affinis® double pegged glenoid prosthesis. Glenoid reaming was planned visually by the surgeon using the patient’s preoperative computer tomography (CT) scan. The aim in a glenoid with retroversion (Walch B type) was to have less than 10^0^ retroversion and > 80% glenoid component seating. The appropriate size glenoid prosthesis was then cemented into position with pressure injected cement. The proximal humerus was then prepared. After trial implantation the definitive stemless humeral prosthesis and ceramic head was then impacted into position. Finally, the shoulder was reduced and the subscapularis reattached with a solid transosseous suture repair. Movement range and stability of the replacement were then recorded. The deltopectoral interval and skin were then closed.

All patients used a sling for 5 to 6 weeks outdoors. A physiotherapy supervised passive motion home exercise program commenced on the first postoperative day. Active ROM was started slowly after 10 weeks, but resisted subscapularis strengthening was deferred until 3 months postoperatively.

### Collected variables and adverse events

Pre-operatively patients had the American shoulder and elbow surgeons (ASES) score [11], Constant score (CS) [12, 13], Shoulder pain and disability index (SPADI) [14], Disability of the arm, shoulder and hand (DASH) scores [15, 16], Pain (VAS 0 = no pain-10 = worst pain imaginable) recorded by independent orthopaedic registered nurses (ORN). Clinical range of motion (ROM) was assessed by a surgeon observed and recorded by an (ORN). At 1 and 2 years post operation radiology assessment, CS, DASH, ASES, SPADI and ROM had been recorded by an orthopaedic surgeon and ORN. The protocol of this study was that after the 2-year clinical review, experienced university research staff and ORNs coordinated annual further follow up, usually by telehealth, and independent orthopaedic surgeon review of recent annual X-rays. At 5 years all 74 patients had final follow-up. ASES, SPADI, VAS pain and satisfaction (scored from 0% - dissatisfied to 100% - totally satisfied) and an independent interpretation of radiology were recorded at this time point.

If any patient, when contacted post operatively, reported an increase in pain or decreased satisfaction, or on X-Ray had a Lazarus score over 1, clinical re-evaluation was organized to elucidate the source of the pain or dissatisfaction. This occurred in 12 patients. Clinical parameters and PROMs including CS score, ASES, SPADI, DASH VAS and ROM were re-assessed after any treatment. Findings were then documented as the patient’s latest score.

Eleven patients were re-assessed at 5 years and included in the 5 year cohort. Seven had received treatment for AC joint pain and 4 for neck pain or chronic pain syndrome. One patient underwent a revision at 4 years for glenoid loosening. This adverse event related to the medical product was reported to the product company and the Australian Orthopaedic Association National Joint Replacement Registry (AOANJRR).

### Radiological assessment

Radiological assessment occurred at 1-day post-surgery, 12 weeks, and yearly thereafter to minimum 5 years. For humeral and glenoid sided assessment, radiographs were taken according to a standardized protocol in multiple planes (axillary, true lateral, standard antero-posterior and true anterior-posterior view of glenoid with the arm in 20° external and internal rotation). Radiographs were assessed independently and separately by two orthopaedic surgeons (MJKS and SH) not involved in the patient surgeries. Disagreements were referred to a third independent experienced surgeon (HC) for final decision.

**Fig. 1:**
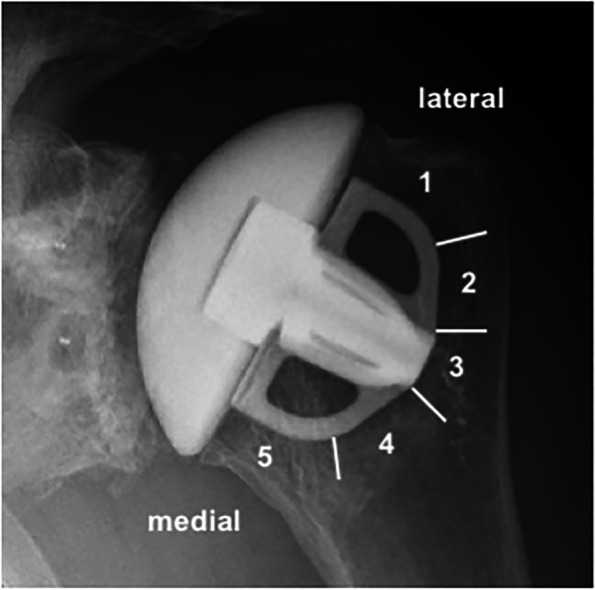
A radiograph demonstrates the humeral component of the prosthesis. The five zones were assessed for radiolucent lines around the humeral component or osteolysis at around the humeral head component

A standard technique for the assessment of peri-humeral humeral component osteolysis, with assessment of five zones (Fig. 1), was used in the radiographical evaluation, as previously described [9]. The glenoid component osteolysis was assessed by quantifying radiolucent lines (RLL) between the glenoid cement/ bone interface around the 2 pegs using the Lazarus score [17].

### Statistical methods

The approach taken has been to consider the precision of the estimated probability of failure of the prosthesis over a specific period together with the possibility of recruiting the specified number of patients within a reasonable time (years). These considerations led to a sample size of about 80 being considered appropriate, and achievable. All analyses were conducted using the statistical package Minitab (Minitab 18 Statistical Software (2017). State College, PA: Minitab, Inc. (www.minitab.com)). Inter-rater reliability for radiograph assessment was evaluated using percentage agreement and Cohen’s kappa interpretation scales. In addition to the standard descriptive statistics of variables, both parametric (Pearson correlation, t-tests and ANOVA) and non-parametric methods (Spearman correlation, Sign test and Kruskal-Wallis test), were used to assess associations between variables. Non-parametric methods were mainly needed to deal with analyses involving the year 2 values of pain and patient satisfaction, as both of these variables were very highly skewed with a majority of values being 0 and 100%, respectively, but, for consistency, they were also used for all of preoperative to year 2 comparisons.

In all cases the results of the parametric and non-parametric tests were essentially the same and, for simplicity, only the results of the non-parametric methods have been reported.

## Results

### Participants and preoperative data

**Fig. 2:**
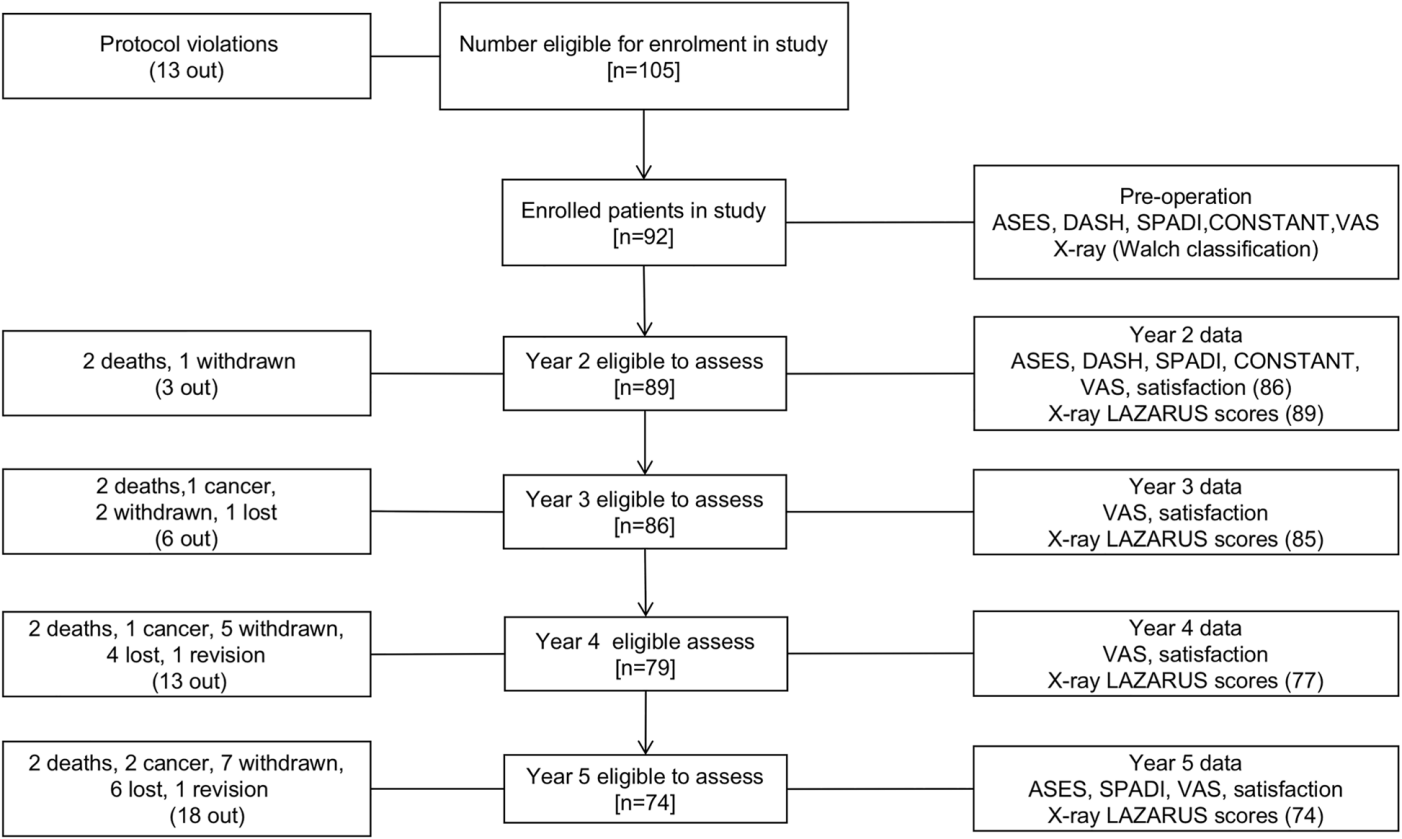
The CONSORT flowchart of the study. Eighteen patients were unavailable at the 5-year follow-up. Two deaths not related to shoulder surgery occurred. Two patients had cancer and were not able to follow protocol. Seven patients withdrew from the follow-up process. Four of these withdrew due to increased age and the other three named no reasons for withdrawal. Six were completely lost to follow-up (potentially moved interstate). One case required a revision from the aTSR to a reverse shoulder replacement and therefore was excluded from the follow-up

Of 105 patients available for the study, 92 were eligible to participate. Thirteen patients had protocol violations including lost and incomplete follow-up (e.g. 2 had died (not related to the prosthesis), 2 had developed cancer). Therefore 74 patients were available for evaluation at 5-year follow-up (Fig. 2). The average age at operation was 68.1 ± 7.1 years (Table 1). The preoperative recorded radiographic Walch classification evaluation score of glenoid wear on CT scan demonstrated 49 type A, 40 type B, and 3 type C, however on retrospective analysis the 3 type C were reclassified as B3 in the new classification [18]. There were no intraoperative fixation problems inserting the humeral prosthesis.

**Table 1:** Patient demographics (*n* = 92) and preoperative radiographic glenoid scores (Walch score) included in this study

	Preoperative patients (***n*** = 92)
Age at operation ± SD	68.1 ± 7.1
Gender	
Female	50
Male	42
Operated Shoulder	
Right	42
Left	50
Dominant Arm	
Right	80
Left	11
Both	1
BMI ± SD	29.9 ± 7.9
Walch classification	
A1	30
A2	19
B1	20
B2	20
C (B3)	3

### Postoperative follow-up

At 2 years there were 89 (96.7%) patients available for assessment. Clinical scores (ASES, CS, DASH, SPADI) were assessed for 86 and radiologic evaluation on 89.

Twelve patients, who reported a clinical deterioration post operatively or had a Lazarus of > 1, were clinically re-evaluated at 5 years with all PROMs, CS, ROM and the results rescored as the latest clinical assessment. In 11 of those 12 patients clinically, there was no evidence of pain related to the prosthesis and radiology confirmed this. Clinical assessment demonstrated that in 7 of the 11 patients the pain was related to the AC joint. In 3 of the 11 patients the pain was assessed as neck pain or chronic pain syndrome. One of the eleven patients had no pain or loss of movement but had a Lazarus score of 2. One patient of the twelve had a serious adverse event and required revision of the prosthesis because of glenoid loosening of the medical device.

The progress of the patients and reasons for incomplete follow up in some are documented in Fig. 2. After 5 years, there were 74 (80.4%) patients in the study remaining for re-evaluation. Six cases were completely lost to follow-up. We were informed that 12 further patients with incomplete follow up, which was related to death or their medical condition, did not require a revision of their aTSR.

The post-operative 2-year clinical PROMs, CS and ROM assessment of the patients improved significantly (*p* < 0.001) (Table 2). Approximately half of the patients had returned to light activities (e.g. gardening), and about 20% of patients could perform medium (e.g. tennis) and 30% heavy (e.g. chainsaw) exercises /activities, respectively. The ASES, SPADI and pain scores continued to improve significantly (*p* < 0.001) and satisfaction levels remained on average over 95% at the 5-year follow-up (Table 2). The improvement in the ASES and pain scores at 5 years was well above the published minimal clinically important difference (MCID) for these scores [19]. Out of the 92 patients enrolled in the study there was one failure/revision, so that the estimated probability of failure within 5 years of surgery is 0.0109 (1/92) with 95% confidence interval (0.0003, 0.0591).

**Table 2:** Clinical assessment preoperatively and with a 2-year follow-up mark and a further 5-year follow-up for ASES, SPADI, pain and satisfaction scores

	Preoperative (n = 92)		Year 2 (***n*** = 86)		Year 5 (***n*** = 74)	
	Mean ± SD	(min, max)	Mean ± SD	(min, max)	Mean ± SD	(min, max)
**Pain (0–10)**	5.56 ± 2.31	(1, 10)	0.44 ± 0.99 ***	(0, 5)	0.41 ± 1.15 ***	(0, 6)
**ASES**	41.62 ± 17.47	(5, 80)	87.34 ± 13.82 ***	(22, 100)	94.28 ± 7.44 ***	(70, 100)
**SPADI**	62.93 ± 18.92	(23.8, 97.7)	5.04 ± 7.38 ***	(0, 37)	4.27 ± 5.96 ***	(0, 26.2)
**DASH**	46.50 ± 16.52	(11, 95)	8.38 ± 11.37 ***	(0, 61)		
**CONSTANT**	30.34 ± 13.35	(5, 66)	77.93 ± 13.31 ***	(42, 96)		
**Satisfaction (%)**			97.62 ± 0.73	(75, 100)	95.71 ± 14.25	(0, 100)
**Active Elevation (**°**)**	91.29 ± 29.95	(10, 180)	151.10 ± 23.23 ***	(80, 180)		
**External Rotation (**°**)**	26.42 ± 19.32	(0, 90)	61.58 ± 16.98 ***	(20, 90)		

(Sign test: * *p* < 0.05; ** *p* < 0.01; *** *p* < 0.001)

### Radiologic assessment

Inter-rater interpretation of the radiographs demonstrated in both evaluations - percentage agreement and Cohen’s kappa – “almost perfect” scores as the inter-rater agreement was 93%. Radiological evaluation of the glenoid component demonstrated good results throughout the five-year follow-up period. Radiolucent lines for the glenoid component did show a minor tendency to increase over the 5-year period. At 5 years there was just one case (1.3%) out of 74 with a Lazarus score of 2 (≤ 2 mm around one peg only), 11 (14.9%), with a Lazarus score of one, leaving 62 (83.8%) with a zero score. The one patient with Lazarus score of 2 had a significant fall upwards boarding a train, with humeral pain and bruising and sternal injury more than 4 years after surgery. It is unclear whether this contributed in the increase of score from 1 on previous annual X-ray to 2 post fall. This score was stable at 5 years. There were no cases with a Lazarus score of 3 or 4.

**Fig. 3:**
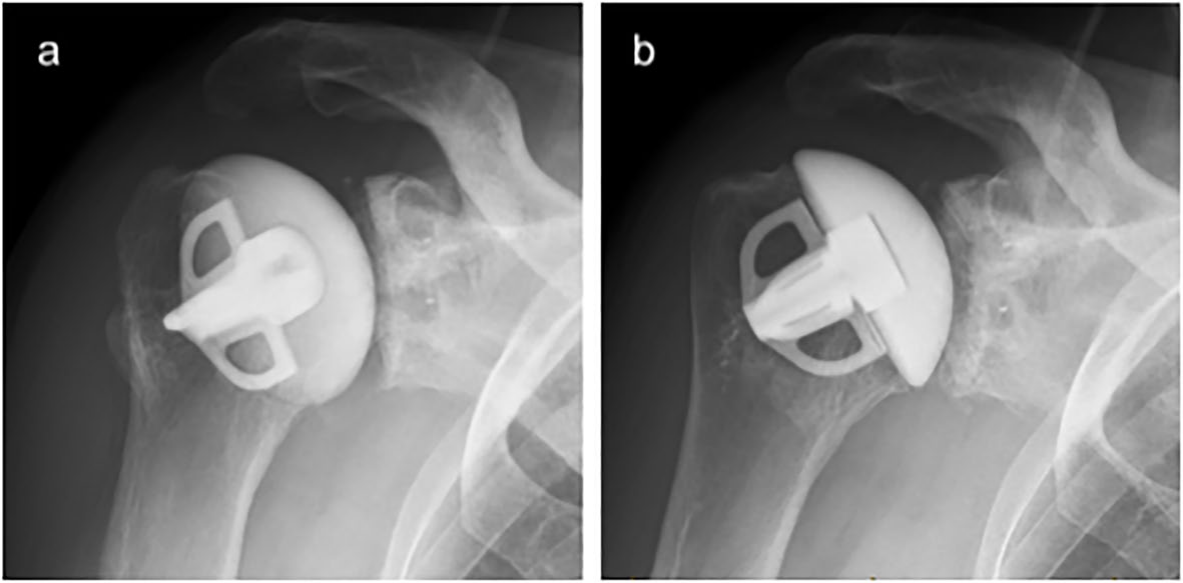
X-ray images, part a and b (true ap with the arm in 20° internal and external rotation, respectively), demonstrate the importance of multiple angles in order to properly assess the glenoid and the humeral component of the prosthesis. Glenoid component radiolucency is graded by the Lazarus score [17].

Radiological assessment of the humeral prosthesis demonstrated at the 5-year follow-up that there were no radiolucent lines identified around the humeral stem component for all zones (Fig. 1 and Fig. 3). At 5 years there were 3 patients, of the 74 eligible, with minor osteolysis in humeral zone 1 (2 cases with 1 mm, 1 with 2 mm) and/or 1 minor osteolysis registered for zone 5 (1 case with 1 mm). No correlation was noted between the radiologic scores (Lazarus glenoid score or the humeral zones) and the pre-operative Walch classification. There also was no evidence that the Walch classification results were correlated with any PROMs, ROM or satisfaction.

No associations were found in the 5-year postoperative PROMs between age, gender, or arm dominance. Higher satisfaction levels with the aTSR were significantly correlated with lesser pain scores at the 5-year follow-up (Kruskal-Wallis (*p* < 0.001)).

### Complications

Throughout the 5-year follow-up period there were a few complications. There were no intraoperative complications particularly in regard to humeral or glenoid component placement. No complications were reported in the early postoperative stage. No infections, fractures or instability episodes were identified for the early and late postoperative follow-up. The eleven cases of 92 (11.9%) with non-prosthesis related postoperative issues, and the one (1.08%) with issues involving the prosthesis, were identified and recorded (Table 3). There were 7 cases (7.6%) with acromioclavicular (AC) joint pain (Table 3). All seven received a cortisone injection into the AC joint. On review, in four patients (4.3%) pain resolved completely. Two patients’ AC joint (2.1%) remained symptomatic and underwent a successful arthroscopic distal clavicle resection. In one case (1.0%) the pain resolved initially then returned; however the patient declined further treatment for the AC joint problem.

**Table 3:** Adverse events of acromioclavicular joint (ACJ) pain and revision surgery. Age (years), gender (M = male; F = female) and if operated arm was the dominant arm (R = right; L = left). It demonstrates actions undertaken, at what time the pain resolved or if an operation was performed and if the issue was resolved

Events	Patient details	Time from Operation	Action	Resolved
**AC Joint pain**	female, non-dominant arm L	55 weeks	steroid injection, resolved at 3 weeks	Yes
	male non-dominant arm L	5 years	steroid injection, resolved at 4 weeks	Yes
	female, dominant arm L	5 years, 10 months	failed steroid injection, resection, pain resolved at 12 weeks	Yes
	female, dominant arm R	40 weeks	steroid injection, resolved at 4 weeks	Yes
	male, dominant arm R	1.5 years	steroid injection, resolved at 4 weeks	Yes
	female, non-dominant arm L	3.5 years	steroid injection, unsustained improvement, mobility, comorbidities, patient chose not to undergo further surgery	No
	female, non-dominant arm R	26 months	failed steroid injection, resection, pain resolved at 9 weeks	Yes
**Revision**	female, non-dominant arm L	3.5 years	Pain with glenoid loosening, Revision to reverse shoulder replacement	Yes

Of the other 4 cases (4.3%), one had Lazarus 2, but had no pain and good function. Three others (3.2%) had diffuse pain not related to the prosthesis, and were thought to have pain of neck origin, perhaps with a chronic pain problem.

There was one cases (1.08%) requiring revision due to early glenoid loosening (Table 3). After 3.5 years, this patient had persistent pain. Although the Lazarus score on X-rays was only 1, on a CT scan lucent lines were more evident around both pegs. There were no signs of infection, rotator cuff tear or other possible reasons for the pain identified. Revision surgery was performed to a reverse TSR. During surgery, no visible wear of the glenoid face or humeral head was identified. The glenoid component was loose with fragmented cement and soft bone and scar tissue around the backside of the glenoid. The stemless humeral component was solid with good bony ingrowth.

## Discussion

This 5-year mid-term follow-up evaluated clinical and radiographic results of this anatomic stemless total shoulder replacement demonstrated that 12 out of 74 cases had a minor radiolucency around the glenoid component (11 - Lazarus 1 and one - Lazarus 2) and significantly improved clinical scores at 5-year follow-up.

In most reports anatomic replacement to treat glenohumeral osteoarthritis with an intact rotator cuff results in pain reduction and improved functionality [20–23]. In the past an aTSR consisted of a long-stemmed humeral stem prosthesis with a metal humeral head articulating with a polyethylene glenoid component. Several stemless aTSR options are now available, although only the Affinis® has a ceramic head option [24]. A direct comparison of our results with other studies of stemless prostheses is difficult, as most other studies have either a shorter follow-up, a combination of both total and hemi arthroplasty, compare different prosthetic bearing surfaces, or different patient demographics, in particular varied pathology requiring replacement rather than exclusively osteoarthritis as in this study [2, 10, 22, 25–27]. There is a study by Spranz et al. comparing a different stemless prosthesis versus a stemmed aTSR in a total of 37 cases of glenohumeral OA with a follow-up ranging from 4.3–6.3 years [28]. There was no radiographic analysis. Their main focus was a 3D-motion-analysis, which demonstrated good ROM for both prosthesis types with no significant differences between groups. However, their stemless group (*n* = 12) had slightly lower external rotation (ER) 40.3° ± 13.9° and active elevation (AE) 126.2° ± 28.5° than the presented cases of our current study (Table 2).

Our current results demonstrate significant reduction of pain scores from pre-operative to 5 year post-operative scores. This is confirmed in other reports on stemless aTSR [2, 22]. Active elevation (AE) and external rotation (ER) improved significantly in the present study, with an AE of 151.10° ± 23.23° at 2-year follow-up resulting in AE above 120° present in 86% of our cases. Gallacher et al. used a stemless humeral component and glenoid resurfacing for patients with a similar age to ours and only cases with osteoarthritis. They reported a little less AE 132° in their two-year follow-up than in our series [21]. Krukenberg et al. used a stemless humeral protheses with a metal head, together with 4 different types of cemented glenoid component, both keeled and pegged. They reported at 2 years a little less AE of 144° ± 30°compared with our study [29]. Razmjou, in another study, compared a stemless aTSR and two different stemmed aTSR. The ROM outcomes were similar between the prostheses, but the stemless design resulted in less radiolucencies or component loosening for the glenoid and humeral components [30]. Their results, combined with those presented here by us, and the in vitro data by Mueller et al. [6] identifying less glenoid polyethylene wear when articulating with a ceramic than a metal head, suggests that current good results of a stemless aTSR coupled with a ceramic head is related to both the stemless design, and the better wear properties of the ceramic articulation.

All postoperative PROMs in this study achieve MCID [19, 31]. The 5-year ASES score increased from the preoperative scores (41.62 ± 17.47 to 94.28 ± 7.44). These ASES scores are similar to Athwal et al., who, using a stem free system, registered preoperative ASES values of 20.48 ± 11.43 and achieved within a 2-year follow-up ASES scores of 89.37 ± 13.26 [26]. The current study results of the Constant score demonstrate an increase from around 30 points pre-operatively to 77.9 points post-operatively similar to the results achieved in the literature by Spranz et al. and Heuberer et al. with a Constant score of 67.9 ± 12.0 and 78.7 ± 15.7 with a mean follow-up of 4.3 and 4.8 years respectively [28, 32]. However, direct comparison with these papers is limited as the studies used varied prosthetic components, or even hemi shoulder arthroplasty, and group size or follow-up was limited [26, 28, 32].

The most common post-operative problem encountered was ACJ-related pain [33]. This is perhaps to be expected as with good shoulder function the often arthritic AC joint is stressed and can become symptomatic.

The radiographic assessment of the glenoid component demonstrates excellent results after 5 years. In the majority of cases (*n* = 62; 83.8%), there were no radiolucent lines visible. Only one case (1.3%) had a Lazarus score of 2 (≤ 2 mm around one peg only), eleven patients (14.9%) had a Lazarus score of one, and only one of these eleven cases required revision for glenoid component loosening. Computer tomography can be more sensitive in assessing radiolucent lines around a cemented pegged glenoid component [34]. Computer tomography in this study was reserved for cases where patient symptoms and plain x-rays did not correspond, in order to avoid increased radiation exposure and cost [34, 35].

The status of the glenoid component fixation is usually the deciding prosthesis factor related to longevity of an anatomic TSR. Radiolucent lines around the glenoid component of a TSR were reported by Roche et al. to not only indicate glenoid loosening but they were also associated with poorer clinical outcomes and higher complication rates [35], which accords with our minor radiolucent lines and good clinical outcomes.

Parks et al. had similar clinical results demonstrating significant improvements in ROM, ASES and pain scores with the use of a cemented hybrid glenoid component and a stemmed humeral prosthesis [36], with an average follow up of 28.7 months. They had a glenoid component loosening of 18.4% (Lazarus scores ranging from 2 to 5) and 7 of the 80 cases had to be revised. Similar to our findings they could not identify a statistical association between the preoperative Walch classification and follow-up radiolucencies or glenoid loosening.

Gallacher et al. demonstrated 8 cases (8%; out of 100 cases) of complete humeral radiolucent lines in one of the 3 humeral grading zones, including the critical zone, for their humeral stemless prosthesis type [21]. There were no cases of similar humeral stem radiolucent lines identified in the current study.

In a study of the same prosthesis as in this current study, Jordan et al. report the results in 279 patients [10]. They had a mixture of total and hemi arthroplasty cases, and varied pathology, rather than just osteoarthritis. Two hundred and seven were analysed, of which 10 required revision during the 4 year follow up. The adjusted CS was the only follow up clinical assessment, so a comparison of PROMS, pain, satisfaction, and ROM with our study is not possible. Their adjusted CS of the 150 cases in their osteoarthritic group was 74.3 which was a little less than our non-adjusted CS of 77.3. They did not comment on the MCID of their results. They demonstrated, as we did, relatively minor osteolysis on both sides of the joint. Of their ten revisions they did have one case of glenoid loosening leading to revision, as in our series.

In a previous study by Bell et al., a direct comparison of the same glenoid component as in the current study was examined in which the glenoid component articulated with a metal-head long-stem prosthesis in 39 shoulders versus the same ceramic stemless humeral prosthesis as in this current study in 23 shoulders [7]. With this 5 year follow up the authors demonstrated significantly more humeral osteolysis in the metal-head group, which also correlated with a significant increase in glenoid peg radiolucent lines [7].

The stemless concept enables improved positioning of the humeral component on the humerus, and also creates different stress on the humeral bone. The data by Mueller et al. [6] identified less glenoid polyethylene wear when articulating with a ceramic than a metal head. This suggests that current good results of a stemless aTSR coupled with a ceramic head are related to both the stemless design, and the better wear properties of the ceramic articulation.

In the future, further reduction of osteolysis can potentially be achieved with other glenoid materials such as vitamin E-enhanced cross-linked polyethylene which has been shown in vitro to result in less polyethylene wear of the glenoid component [37]. Such a prosthesis is now routinely used, but was not available during this current study.

A limitation of the study is that there is no control arm, such as the use of the same stem with a metal head. However, following the results of previous studies [6, 7], we consider it would be difficult to gain support, funds, and ethics to conduct a study of a metal head on a stemless prosthesis, as there appears to be no clinical disadvantage of using a ceramic head such as fracture, and with a theoretical advantage of better wear on polyethylene [6]. Another limitation is that in the protocol at 2 year follow up all PROMs, CS and ROM were assessed with radiology. However, at 5 year follow up the PROMS of ASES and SPADI, pain, and satisfaction were assessed. This protocol was set up to accommodate geographical considerations for many country patients.

## Conclusion

This study demonstrates that a stemless aTSR with a ceramic head and pegged cemented polyethylene glenoid component resulted in at 5 year follow up overall good clinical scores, little osteolysis, and few complications.

## Data Availability

The datasets used and/or analysed during the current study are available from the corresponding author on reasonable request. Individual data are not publicly available due to data containing information that could compromise research participant privacy/consent.

## Abbreviations

ACJ: Acromioclavicular joint
AE: Active elevation
ANOVA: Analysis of variance
ASES score: American shoulder and elbow surgeons score
aTSR: Anatomic total shoulder replacement
CS: Constant score
CT: Computer tomography
DASH score: Disability of the arm, shoulder and hand score
ER: External rotation
MCID: Minimal clinically important difference
ORN: Orthopaedic registered nurses
PROMs: Patient-reported outcome measures
RLL: Radiolucent lines
ROM: Range of motion
SPADI: Shoulder pain and disability index
VAS: Visual analogue scale (0 = no pain-10 = worst pain imaginable)
